# Insights into the molecular underlying mechanisms and therapeutic potential of endoplasmic reticulum stress in sensorineural hearing loss

**DOI:** 10.3389/fnmol.2024.1443401

**Published:** 2024-12-18

**Authors:** Guanzhen Li, Huiming Yang, Peiyuan Zhang, Yan Guo, Lili Yuan, Shujiao Xu, Yingxue Yuan, Huabao Xiong, Haiyan Yin

**Affiliations:** ^1^School of Clinical Medicine, Jining Medical University, Jining, Shandong, China; ^2^Department of Otolaryngology-Head and Neck Surgery, Shandong Provincial Hospital Affiliated to Shandong First Medical University, Jinan, China; ^3^School of Basic Medical Science, Jining Key Laboratory of Pharmacology, Jining Medical University, Jining, Shandong, China; ^4^School of Dental Medicine, Jining Medical University, Jining, Shandong, China; ^5^Department of Pathology, Shandong Provincial Hospital Affiliated to Shandong First Medical University, Jinan, China; ^6^Institute of Immunology and Molecular Medicine, Jining Medical University, Jining, Shandong, China

**Keywords:** sensorineural hearing loss, endoplasmic reticulum stress, unfolded protein response, oxidative stress, apoptosis, inflammation, autophagy

## Abstract

Sensorineural hearing loss (SNHL) is characterized by a compromised cochlear perception of sound waves. Major risk factors for SNHL include genetic mutations, exposure to noise, ototoxic medications, and the aging process. Previous research has demonstrated that inflammation, oxidative stress, apoptosis, and autophagy, which are detrimental to inner ear cells, contribute to the pathogenesis of SNHL; however, the precise mechanisms remain inadequately understood. The endoplasmic reticulum (ER) plays a key role in various cellular processes, including protein synthesis, folding, lipid synthesis, cellular calcium and redox homeostasis, and its homeostatic balance is essential to maintain normal cellular function. Accumulation of unfolded or misfolded proteins in the ER leads to endoplasmic reticulum stress (ERS) and activates the unfolded protein response (UPR) signaling pathway. The adaptive UPR has the potential to reestablish protein homeostasis, whereas the maladaptive UPR, associated with inflammation, oxidative stress, apoptosis, and autophagy, can lead to cellular damage and death. Recent evidence increasingly supports the notion that ERS-mediated cellular damage responses play a crucial role in the initiation and progression of various SNHLs. This article reviews the research advancements on ERS in SNHL, with the aim of elucidating molecular biological mechanisms underlying ERS in SNHL and providing novel insights for the treatment.

## 1 Introduction

Sensorineural hearing loss (SNHL) is one of the most common types of hearing loss (HL), which affects social interactions and increases the likelihood of developing dementia and depression (Li et al., 2014; Mener et al., 2013; Livingston et al., 2020). SNHL is mainly caused by the impairment of non-renewable hair cells (HCs) and spiral ganglion neurons (SGNs) in the inner ear. Congenital gene mutations (Lv et al., 2021), prolonged noise exposure (Guo et al., 2021; Liberman, 2017), utilization of ototoxic medications (He et al., 2017; Lang et al., 2005; Liu et al., 2019), and aging (Bao and Ohlemiller, 2010; He et al., 2021; Kujawa and Liberman, 2015) are the major risk factors for SNHL. Hitherto, the specific pathophysiologic mechanisms of SNHL remain unclear. Since there is currently no effective treatment for almost all SNHL, patients can usually only rectify their hearing impairment through hearing aids and cochlear implants, but the patient experience is not ideal (Giraudet et al., 2018; Petit et al., 2023; Ferguson et al., 2017). Although different species of SNHL have distinct pathophysiologic mechanisms, their potential mechanisms often involve inner ear gene mutations, oxidative stress, apoptosis, inappropriate inflammation, and autophagy (Wong and Ryan, 2015). Accumulated data indicate that endoplasmic reticulum stress (ERS) plays a role in the above cellular processes and is involved in a variety of disease processes (Zhang et al., 2024). Recently, more and more studies have been conducted on the role of ERS in various types of SNHL, indicating that ERS plays a role in SNHL, which has potential therapeutic benefits for predicting pharmacological manipulations targeting ERS (Wu J. et al., 2020).

The endoplasmic reticulum (ER) in eukaryotes is an organelle comprising a continuous membrane; it is responsible for the processing, folding, and transportation of secreted and membrane-bound proteins, as well as the regulation of intracellular Ca^2+^ concentrations and formation of cell membrane lipids (Schwarz and Blower, 2016). It is believed that glucose deficiency, abnormal calcium regulation, hyperglycemia, hyperlipidemia, viral infections, and hypoxia contribute to the production and accumulation of unfolded/misfolded proteins (UFP/MFP), which leads to ERS (Fedoroff, 2006; Feldman et al., 2005; Fonseca et al., 2011; Görlach et al., 2006; Iurlaro and Muñoz-Pinedo, 2016; Kaufman et al., 2002; Sawada et al., 2008; Zhang and Wang, 2012). To restore ER homeostasis, the adaptive unfolded protein response (UPR) is activated (Hetz et al., 2020). However, when ERS is severe, oxidative stress, apoptosis, inflammation, and autophagy downstream of maladaptive UPR are activated and amplified, leading to cell damage or death (Iurlaro and Muñoz-Pinedo, 2016). It has been identified that ERS-induced cell death was involved in a variety of diseases, including neurodegenerative disorders (Duran-Aniotz et al., 2017; Valdés et al., 2014), cardiovascular ailments (Ren et al., 2021), and liver damage disorders (Zhang et al., 2022). In addition to the previously mentioned areas, research has highlighted the critical role of ERS in various forms of SNHL, including genetic, drug-induced, age-related, and noise-induced SNHL (Jia et al., 2018; Wen et al., 2021; Lee et al., 2021). The involvement of ERS across these diverse types of SNHL suggests its widespread and significant influence in this domain, although comprehensive reviews detailing the entire underlying mechanism remain lacking. Therefore, this review aims to elucidate the mechanisms and identify potential novel therapeutic targets of ERS in inner ear cells, hereby enhancing our understanding the role of ERS in SNHL and offering new perspectives for the treatment.

## 2 Molecular mechanisms underlying ERS

**Figure 1:**
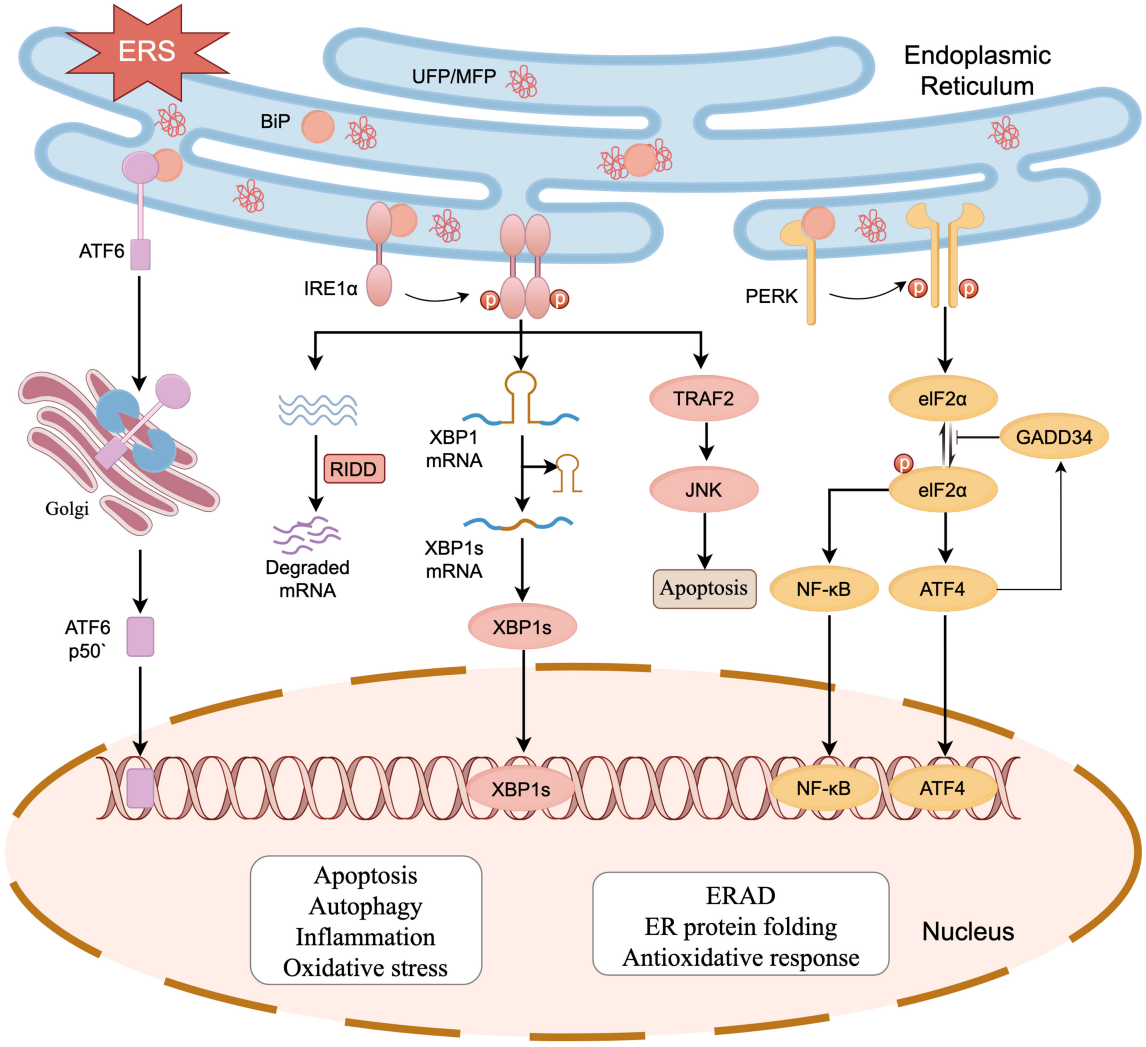
The UPR initiated by ERS in SNHL. Activation of the UPR is regulated by three transmembrane protein receptors: PERK, IRE1α, and ATF6. When UFP/MFP accumulates in the ER, BiP dissociates from PERK, IRE1α, and ATF6, after which it participates in the processing of UFP/MFP. PERK and IRE1α are activated by dimerization and phosphorylation. PERK phosphorylates eIF2α reducing protein translation. IRE1α exerts nucleic acid endonuclease activity and splices XBP1 mRNA. Meanwhile, IRE1α also breaks down a segment of mRNA via a biological mechanism known as regulated IRE1-dependent decay (RIDD), reducing the protein burden in the ER. ATF6 is spliced into ATF6P50 by the Golgi apparatus to exert its biological activity. The three activated receptor proteins also start the transcription of UPR in the nucleus. The cell damage and death responses downstream of UPR (e.g., oxidative stress, apoptosis, inflammation, and autophagy) can act on HCs, SGNs, and stria vascularis, which in turn leads to SNHL (figure drawn by Figdraw).

The key process of ERS is the UPR, which is mediated mainly by three transmembrane protein receptors located on the ER: pancreatic endoplasmic reticulum kinase (PERK), inositol-requiring enzyme 1α (IRE1α), and activating transcription factor 6 (ATF6). Typically, these three proteins combine with the heat shock protein 70 family member BiP (also referred to as GRP78) to maintain ER homeostasis. When ER homeostasis is disrupted, leading to ERS, BiP dissociates from its receptor proteins and binds to UFP/MFP, triggering oligomerization and phosphorylation of the three UPR receptor proteins (Ron and Walter, 2007). Cumulative UFP/MFP is degraded by ubiquitination via the endoplasmic reticulum-associated degradation (ERAD; Hetz et al., 2015). However, the adaptive capacity of the UPR is finite. When ERS is hard to mitigate within the cell, the pro-cell death responses downstream of the maladaptive UPR are activated and amplified, leading to tissue and organ damage (Figure 1; Hetz et al., 2020).

### 2.1 The IRE1α, PERK, and ATF6 signaling pathways

IRE1α, a conserved ERS receptor expressed extensively in the ER membrane, comprises an N-terminal ER lumenal structural domain, a transmembrane region, and a C-terminal cytoplasmic region. The cytoplasmic region contains a site-specific nucleic acid endonuclease structural domain and a serine/threonine-protein kinase structural domain. During ERS, IRE1α detects UFP/MFP via its N-terminal luminal structural domain, separates from BiP and undergoes dimerization and phosphorylation, which activates the RNase structural domain. The activated IRE1α specifically recognizes and cleaves mRNA encoding X box binding protein 1 (XBP1), thereby initiating splicing of XBP1 mRNA to generate an XBP1 protein with transcription factor activity (Calfon et al., 2002; Shen et al., 2001; Yoshida et al., 2001). XBP1 upregulates the expression of genes related to protein transport, folding, secretion, and degradation (e.g., ERAD) in response to ERS and promotes adaptive cell survival (Kaufman, 2002; Yücel et al., 2019). IRE1α also breaks down a segment of mRNA via regulated IRE1-dependent decay (RIDD), thereby reducing the protein burden in the ER (Chen and Brandizzi, 2013). PERK is an ER transmembrane protein belonging to the serine/threonine kinase family and activated by its phosphorylation and dimerization, which in turn leads to phosphorylation of eukaryotic translation initiation factor 2 subunit-α (eIF2α), thereby reducing translation of most proteins and lowering the ER load (Ron and Walter, 2007; Kaufman, 2002). Phosphorylated elF2α promotes the expression of activating transcription factor 4 (ATF4; Blais et al., 2004; Harding et al., 1999). At the early stage of UPR, ATF4 upregulates the transcriptional expression of ER chaperone proteins to restore ER homeostasis. In the prolonged UPR state, ATF4 binds to UPR components and promotes the expression of C/EBP homologous protein (CHOP) to induce ATP depletion, oxidative stress, and apoptosis (Prasad and Greber, 2021). ATF4 also upregulates the expression of growth arrest and DNA damageinducible 34 (GADD34), which inhibits phosphorylation of eIF2α via negative feedback (Hetz et al., 2020). ATF6 is a type II transmembrane protein in the ER. In the case of ER homeostasis imbalance, ATF6 separates from BiP and enters the Golgi via vesicle transport, liberating a 50 kDa basic leucine zipper fragment known as “ATF6p50,” a process facilitated by site-1 protease (S1P) and site-2 protease (S2P). ATF6p50 is transported to the nucleus, resulting in the upregulation of UPR-associated proteins such as XBP1, CHOP, and ERAD components, thereby regulating protein homeostasis in cytoplasm (Haze et al., 1999; Ye et al., 2000; Jin et al., 2017).

### 2.2 ERS and oxidative stress, apoptosis, inflammation, and autophagy

**Figure 2:**
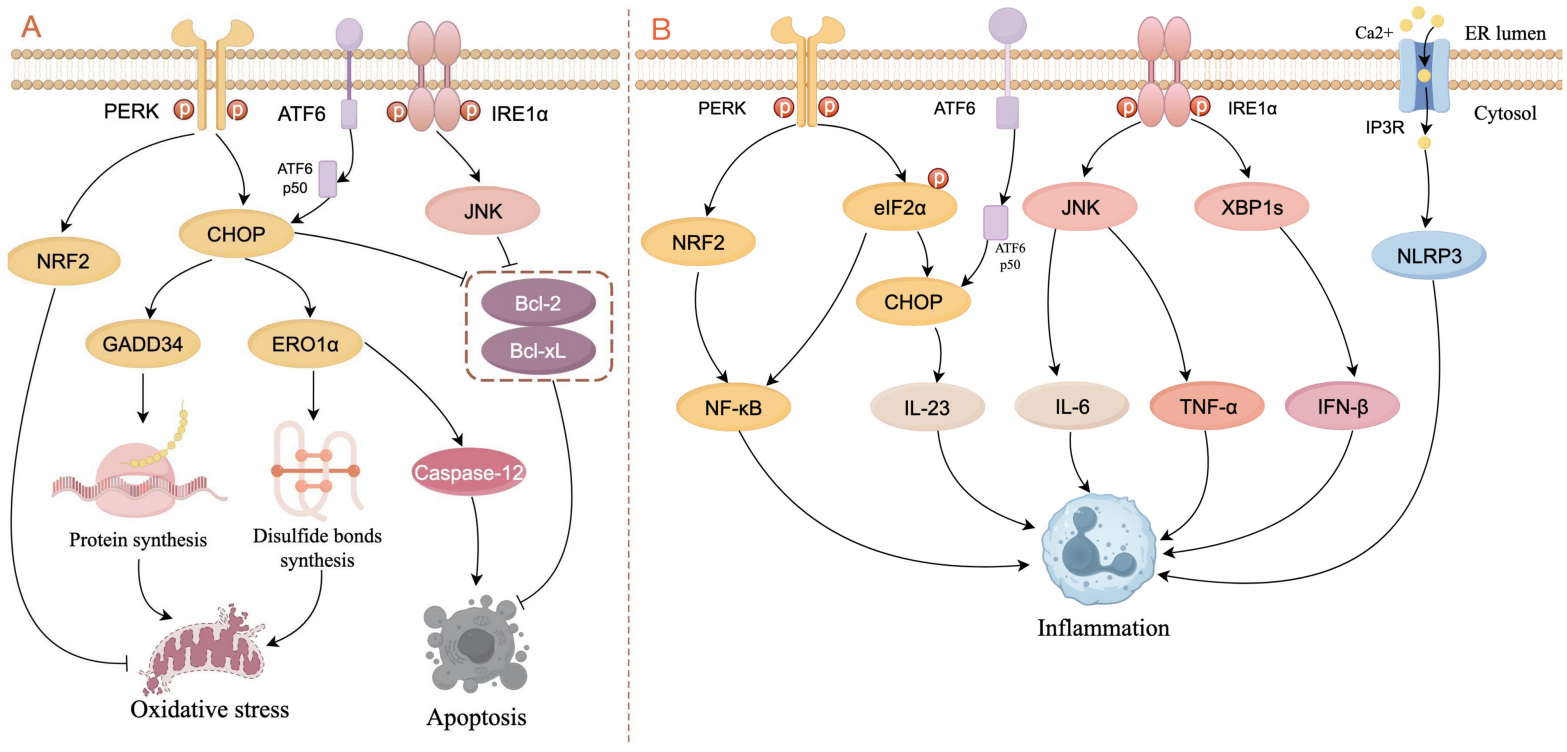
Schematic diagram of ERS signaling in the control of oxidative stress, apoptosis **(A)**, and inflammation **(B)**. **(A)** In the PERK pathway, CHOP activates GADD34 and ERO1α to respectively increase the formation of proteins and disulfide bonds, thereby leading to oxidative stress. CHOP also induces apoptosis by activating caspase-12 or inhibiting the activity of Bcl-2 and Bcl-xL. NRF2 is activated by PERK to inhibit oxidative stress. ATF6p50 increases apoptosis by up-regulating the expression of XBP1 and CHOP through the PERK pathway. The IRE1α/JNK pathway inhibits apoptosis by suppressing the activity of Bcl-2 and Bcl-xL. **(B)** In the PERK pathway, NRF2 and phosphorylated elF2α activate NF-κB to induce inflammation, and CHOP increases the expression of IL-23. In the IRE1α pathway, JNK increases the expression of IL-6 and TNF-α, and XBP1s increase the expression of IFN-β. ERS can result in the opening of the IP3R channel, thereby activating the NLRP3 inflammasome (figure drawn by Figdraw).

An imbalance in redox homeostasis is one of the characteristics of ERS (Ron and Walter, 2007). CHOP can increase the expression of target genes such as ER oxidoreductase 1α (ERO1α) and GADD34, leading to the generation of oxidative stress. GADD34 induces the generation of reactive oxygen species (ROS) by upregulating synthesis of protein (Han et al., 2013; Marciniak et al., 2004). ERO1α is essential for the formation of disulfide bonds, which contribute to protein folding and transfer of electrons to molecular oxygen, thereby promoting the oxidation of ER proteins (Chen et al., 2015; Ramming et al., 2015). In addition, the UPR plays a role in PERK-mediated activation of nuclear factor erythroid 2-related factor (NRF2), which helps maintain glutathione levels, buffering the accumulation of ROS (Cullinan and Diehl, 2004; Cullinan et al., 2003; Figure 2A).

It is widely recognized that ERS-induced apoptotic signaling is mediated mainly by IRE1, PERK, and ATF6, which regulate B-cell lymphoma 2 (Bcl-2) family proteins directly or indirectly to activate intrinsic and caspase-induced apoptotic pathways. CHOP is considered to be an important factor in inducing apoptosis: not only does CHOP inhibit the expression of anti-apoptotic genes such as Bcl-2 (Tabas and Ron, 2011), but also activates the apoptosis-specific molecule caspase-12 in the ER by activating ERO1α, thereby initiating apoptosis activated by other caspase proteins (Gu et al., 2016; Wu H. et al., 2020; Nakagawa et al., 2000). Activated IRE1α recruits and activates the adapter molecule (i.e., TRAF2) of apoptosis signal-regulating kinase 1 (ASK1) and upregulates phosphorylation of c-Jun N-terminal kinase (JNK), which inhibits the expression of Bcl-2 and B-cell lymphoma-extra large (Bcl-xL), thereby promoting apoptosis (Urano et al., 2000; Saveljeva et al., 2015). ATF6p50 increases apoptosis by up-regulating the expression of XBP1 and CHOP through the PERK pathway (Yoshida et al., 2000). Studies have shown that apoptosis caused by overexpression of CHOP and GRP78, as well as activation of caspase-12, is associated with kidney injury (Gao et al., 2014; Kong et al., 2013). In another study in auditory cells, researchers demonstrate that ERS not only activates the apoptotic pathway mediated by caspase-3/caspase-9 but also induces receptor-interacting serine/threonine-protein kinase 1 (RIPK1)-mediated necroptosis (Saveljeva et al., 2015; Kishino et al., 2019; Figure 2A).

ERS and the UPR are involved extensively in inflammatory signaling. PERK activates NRF2 and phosphorylates eIF2α to upregulate the expression of nuclear factor kappa B (NF-κB), thereby inducing inflammation (Deng et al., 2004; Ma et al., 2024). IRE1 binds to the TRAF2-JNK pathway to induce pro-inflammatory cytokine expression (e.g., IL-6 and TNF-a; Kawasaki et al., 2012). Under conditions of ERS, the opening of the Ca^2+^ channel inositol 1,4,5-triphosphate receptor type 3 (IP3R) leads to active transport of Ca^2+^ between the ER and mitochondria, thereby activating the NLRP3 inflammasome (Li et al., 2020). In addition, XBP1 and CHOP stimulate inflammatory cytokine production directly by binding to cytokine promoter and enhancer elements (Goodall et al., 2010; Smith et al., 2008; Figure 2B).

**Figure 3:**
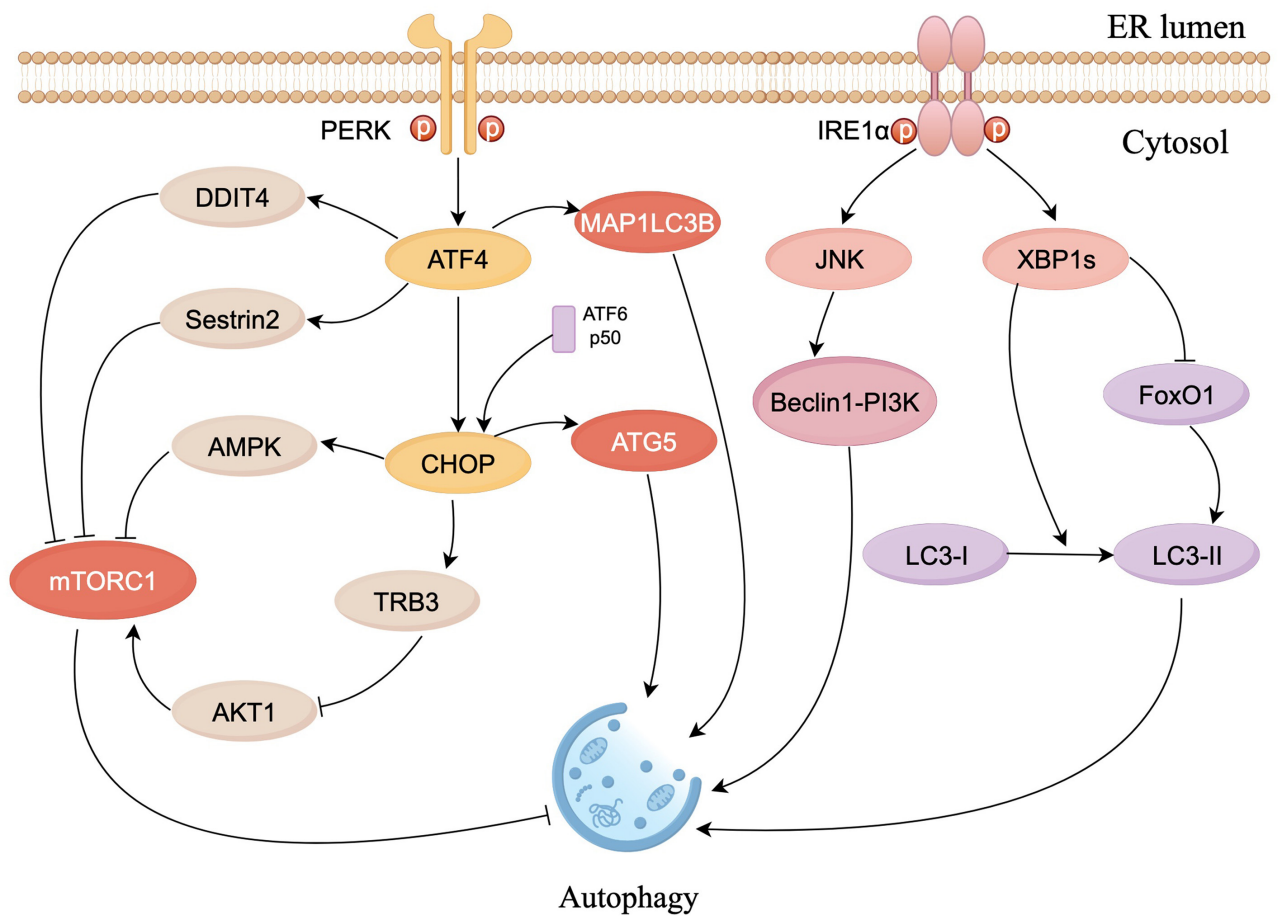
Schematic diagram of ERS signaling in the control of autophagy. The PERK pathway inhibits mTORC1 activity via ATF4 and CHOP to promote autophagy. CHOP constrains the phosphorylation of AKT1 by upregulating tribbles homolog 3 (TRB3) to inhibit mTORC1. ATF4 and CHOP also respectively activate MAP1LC3B and ATG5, to directly induce autophagy. IRE1α/JNK pathway activates Beclin1-PI3K complex to enhance autophagic responses. XBP1s can induce autophagy by facilitating LC3I to LC3II conversion and suppress autophagy by inhibiting FoxO1 (figure drawn by Figdraw).

Autophagy maintains cellular homeostasis in eukaryotes through the degradation and recycling of intracellular biomolecules and damaged organelles (Yang et al., 2016). Autophagy is linked to ERS and regulated by the UPR. Under hypoxia-induced ERS, ATF4, and CHOP increased the expression of microtubule-associated protein 1 light chain 3β (MAP1LC3B) and autophagy-regulated gene 5 (ATG5), thereby promoting autophagy (Rouschop et al., 2010). The PERK pathway induces autophagy by inhibiting the activity of mammalian target of rapamycin complex 1 (mTORC1), a core factor that inhibits autophagy, through the ATF4-mediated expression of Sestrin2 and DDIT4 genes (Brüning et al., 2013; Jin et al., 2009). In addition, PERK can also inhibit AKT1 or activate AMPK through CHOP, thereby inhibiting mTORC1 activity and promoting autophagy (Cybulsky, 2017; Ohoka et al., 2005; Rashid et al., 2015). Meanwhile, eIF2α-ATF4 promotes autophagy by binding to the amino acid response element (AARE) sequence of the p62 promoter (B'chir et al., 2013). In endothelial cells, autophagy is promoted by increasing the conversion of LC3I to LC3II via the IRE1α-XBP1 pathway (Margariti et al., 2013). The IRE1α-JNK pathway phosphorylates Bcl-2, which activates the Beclin1-PI3K complex to enhance autophagic responses (Heath-Engel et al., 2008; Wei et al., 2008). Tunicamycin (an ERS activator) causes splicing of XBP1 mRNA and triggers autophagy, which was confirmed by reports that knockdown of IRE1α, XBP1, and Forkhead box O1 (FoxO1) inhibited the increased expression of LC3-II; additionally the interaction between XBP1 and FoxO1 was confirmed to regulate ERS-induced autophagy in HEI-OC1 auditory cells (Kishino et al., 2017; Figure 3).

Taken together, ERS is widely involved in multiple gene expressions, oxidative stress, apoptosis, inflammation and autophagy processes. Since the specific pathological mechanism of SNHL is still unclear, this suggests that ERS may participate in the occurrence and development of SNHL by communicating with the above biological processes.

## 3 ERS and SNHL

### 3.1 ERS in genetic SNHL

Approximately 1 in 500 newborns are estimated to have congenital hearing loss, and more than half of such cases are attributable to genetic factors (Omichi et al., 2019). However, treatments to reverse or prevent genetic SNHL remain limited. Currently, there are over 250 genes linked to both syndromic and non-syndromic deafness (Carpena and Lee, 2018), and some of these genes and their protein products are tightly associated with ERS.

Usher syndrome (USH) is a condition in which patients with USH experience reduced hearing and congenital SNHL. USH is associated with three USH proteins: Cdh23, scaffold, and Myo7a. The absence of any one of these proteins contributes to the development of severe USH (Blanco-Sánchez et al., 2014; Bonnet and El-Amraoui, 2012). Studies conducted in zebrafish inner ear hair cells (HCs) demonstrate that the three USH proteins form a complex that regulates ER vesicular transportation in the HCs; inhibiting the synthesis of this complex leads to ERS and promotes apoptosis of HCs through the Cdk5- Mekk1-JNK pathway (Blanco-Sánchez et al., 2014; Kang et al., 2012). In addition, the mutation of Cdh23 gene induces apoptosis in HCs and is thought to be a pathological cause in patients with non-syndromic autosomal recessive deafness (DFNB12) and USH type 1D (USH1D; Bolz et al., 2001; Bork et al., 2001; Miyagawa et al., 2012; Han et al., 2012). Studies of a DFNB12 mouse model (erl mouse) in which deafness is caused by a Cdh23 point mutation revealed that expression of BiP and CHOP in outer hair cells (OHCs), SGNs, and stria vascularis increased significantly, and confirmed that apoptosis was inhibited significantly after CHOP knockout (Hu et al., 2016). P4-ATPase is a phospholipid flippase that selectively transports phospholipids from the ectoplasm to the cytoplasmic leaflet to maintain lipid membrane asymmetry (Coleman et al., 2009; Tang et al., 1996; Zhou and Graham, 2009). Deletion of proteins belonging to the P4-ATPase superfamily can lead to hearing disorders (Chepurwar et al., 2023; Coleman et al., 2014; Pater et al., 2022; Stapelbroek et al., 2009). Transmembrane protein 30A (TMEM30A) is one of the most common forms of P4-ATPase. Lack of TMEM30A disrupts HC planar polarity in the cochlea of mice, a process accompanied by an increase in ERS, as detected by increased expression of CHOP and BiP (Tone et al., 2020; Xing et al., 2023). Mesencephalic astrocyte-derived neurotrophic factor (MANF), located in the ER, mainly interacts with BiP to maintain protein folding homeostasis (Glembotski et al., 2012; Mizobuchi et al., 2007; Yan et al., 2019). Research shows that MANF is expressed in HCs and neurons, as well as in some non-sensory cells in the cochlea (Herranen et al., 2020). Meanwhile, in *manf* knockout mice, the OHCs gradually die soon after hearing formation, and expression of CHOP increases, indicating that MANF plays an important role in maintaining hearing by opposing ERS (Herranen et al., 2020). TMCC2 is an ER-resident transmembrane protein and is widely expressed in HCs of the mouse inner ear (Wisesa et al., 2019; Hoyer et al., 2018; Sohn et al., 2016; Zhang et al., 2014; Hopkins et al., 2011; Ren et al., 2023). Researchers performed auditory brainstem response (ABR) measurements on *Tmcc2* knockout mice and showed that TMCC2 deletion leads to congenital HL. Further research has shown that the cause of HL is associated with a progressive loss of HCs. Meanwhile, an increase in ERS was observed in *Tmcc2* knockout HCs, although the general morphology and functions of ER were not affected. This suggests that deletion of TMCC2 would likely lead to auditory HC death through increased ERS (Ren et al., 2023). Gap junctions facilitate intercellular communication and play an important role in maintaining cellular homeostasis (Cohen-Salmon et al., 2002; Simon and Goodenough, 1998). CX31, a significant component of gap junctions, is highly expressed in the inner ear HCs and SGNs of mice. Deficiency or malfunction of CX31 causes SNHL associated with upregulation of the chaperone protein BiP in the UPR pathway in HCs and SGNs (Xia et al., 2010; López-Bigas et al., 2001). This points to a potential connection between hearing impairment resulting from CX31 deficiency and ERS-induced death in the cochleae.

### 3.2 Drug-induced SNHL

#### 3.2.1 Cisplatin-induced SNHL

Cisplatin is a common chemotherapeutic agent used to treat various types of cancer. The introduction of cisplatin into the inner ear triggers a cascade of events, including inflammation, oxidative stress, and DNA damage, leading ultimately to the death of HCs and subsequent SNHL (Qu et al., 2023). Cisplatin-induced apoptosis-associated activation of caspase-3 in the cytoplasm requires calcium, as well as activity of the calcium-dependent protease calpain, which is associated with ERS suggesting that the ER is a potential target for cisplatin (Mandic et al., 2003). Mandic et al. found that cisplatin induces calmodulin-dependent activation of the ER-specific caspase-12 in the cytoplasm, and increases expression of BiP (Mandic et al., 2003). Studies in cultured murine cochlear explants showed that cisplatin increases expression of BiP, CHOP, and caspase-3/9/12 in the inner ear, suggesting that cisplatin-induced apoptosis is associated with activation of the caspase-12 apoptotic pathway downstream of the UPR (Xiao et al., 2016). The expression of ATF4 and CHOP increases progressively in cisplatin-treated hair cell-like OC1 cells, and cisplatin toxicity is reduced significantly in *chop*-knockout cells indicating that the PERK/ATF4/CHOP pathway is involved in cisplatin-mediated ototoxicity (Qu et al., 2023). The protein arginine methyltransferase 3 (PRMT3) and cannabinoid systems are involved in cisplatin-induced ototoxicity associated with ERS, and apoptotic signaling (including caspase-3, caspase-9, poly-adenosine diphosphate-ribose polymerase, and phospho-p53) is enhanced, in HEI-OC1 cells (Lim et al., 2019). Overexpression of PRMT3 or FAAH1 increased apoptosis and ERS signaling, whereas knockdown of PRMT3 or the activation of cannabinoid 1 receptor (CB1R) and inhibition of FAAH1 mitigated cisplatin ototoxicity (Lim et al., 2019). ERS is involved in cisplatin-induced ototoxicity through activation of ER autophagy proteins (Gentilin et al., 2019); indeed, the ER autophagy receptor FAM134B has been shown to promote ER autophagy during ERS (Mo et al., 2020). Increased expression of LC3B in cisplatin-treated HEI-OC1 cells correlates with time-dependent expression of ER autophagy receptor FAM134B (Yang et al., 2023). Knockdown of FAM134B decreases cisplatin-induced autophagy, and attenuates ERS as well as expression of apoptotic factors, suggesting that FAM134B-induced autophagy in the ER is an important pathway in cisplatin-mediated ototoxicity (Yang et al., 2023).

#### 3.2.2 Aminoglycoside antibiotic-induced SNHL

An overdose of Aminoglycoside antibiotic (AmAn) causes significant ototoxicity by inhibiting protein synthesis and inducing ROS in host cells, leading to apoptosis of HCs (Francis et al., 2013; Shulman et al., 2014; Xie et al., 2011). Oishi et al. conducted genome-wide transcriptomic and proteomic analyses and reported a substantial increase in the expression of BiP, GRP94, ATF4, and calreticulin in the gentamicin-treated group (Oishi et al., 2015). Furthermore, gentamicin significantly reduces the number of SGNs in XBP1 haploid mice (Oishi et al., 2015). Tu et al. discovered that chronic kanamycin induces extensive apoptosis in SGNs, which correlates positively with the expression level of caspase-12 (Tu et al., 2019). These studies indicate that SGNs are an important target for AmAn drugs, resulting in ototoxicity via ERS. In addition, Wu et al., found that neomycin induced significant apoptosis in HEI-OC1 cells through the PERK/eIF2α/ATF4 pathway (Wu et al., 2022). Thus, AmAn causes damage to inner ear HCs and SGNs by promoting the ERS pathway, leading to ototoxicity.

#### 3.2.3 Other types of drug-related SNHL

Acetaminophen (APAP) is a non-steroidal anti-inflammatory drug and has ototoxic side effects (Blakley and Schilling, 2008; Curhan et al., 2010, 2012). A metabolite of APAP, N-acetyl-P-benzoquinone imine (NAPQI), causes damage to the ER and triggers ERS (Ramachandran and Jaeschke, 2018; Xiaomeng et al., 2020). Kalinec et al. demonstrated that APAP and NAPQI trigger ERS and ototoxicity in HEI-OC1 cells via the PERK/eIF2α/CHOP pathway, and found that the PERK pathway-mediated cell death pathway was independent of ATF4 (Kalinec et al., 2014). This implies that ototoxicity induced by APAP is a result of ERS-induced death signaling via a different pathway (Kalinec et al., 2014). Pyridoxine, also known as vitamin B6, can cause peripheral neuropathy (Schaumburg et al., 1983). In 2019, Park et al. discovered that treatment of an auditory neuroblastoma cell line with a surplus of pyridoxine resulted in elevated ROS levels, mitochondrial dysfunction, and upregulation of UPR (including p-PERK, GRP78, CHOP, and caspase-12; Park et al., 2019). This indicates that the administration of excess pyridoxine may contribute to the impairment of auditory nerve cells through the ERS-induced apoptotic pathway, potentially leading to SNHL. However, whether apoptosis is directly mediated by ERS still needs to be further verified.

### 3.3 Age-related hearing loss

Age-related hearing loss (ARHL) arises in conjunction with the natural progression of aging, resulting in significant morbidity (Eggermont et al., 2017). Chronic ERS is part of the aging process. Wang et al. found that accumulation of ubiquitinated proteins, as well as reduced expression of BiP and high expression of CHOP and caspase3/9, occurs in the cochlea of mice aged 12–14 months (Wang et al., 2015). Lee et al. conducted *in vitro* and *in vivo* experiments, which revealed that in the cochlea aging causes a substantial reduction in the expression of heat shock proteins, including HSF1, HSP70, and HSP40, all of which are essential for preserving ER balance; in addition, expression of p-eIF2α and CHOP increases as that of HSF1 falls (Lee et al., 2021). These studies suggest that ERS is involved in the progression of cochlear senescence and that strategies aimed at reducing ERS-dependent apoptosis in the aging cochlea could play a role in preventing ARHL.

### 3.4 Noise-induced hearing loss

Noise-induced hearing loss (NIHL) is the most common occupational disease in many Asian countries (Fuente and Hickson, 2011). Previous studies have confirmed that cochlear cells undergo apoptosis and necrosis during the pathogenesis of NIHL, although the exact process is unclear. One study in guinea pigs explored the role of ERS in cochlear damage induced by exposure to intense noise. Both ER-related BiP and CHOP levels in the cochlea were elevated significantly after exposure to noise, accompanied by marked apoptosis, necrosis, and degeneration of OHCs (Xue et al., 2016). Researchers speculated that the ERS response was activated by inducing BiP to mitigate noise-induced damage to cochlear cells and that the CHOP pathway was activated to eliminate the most severely injured cells (Xue et al., 2016). TMTC4 is expressed extensively in the ER and plays a role in regulating Ca^2+^ dynamics and the UPR (Li et al., 2018). Inactivation of the gene *tmtc4* led to the development of NIHL in mice; however, concomitant knockdown of *chop* attenuates HL (Li et al., 2018). These studies demonstrate the direct link between NIHL and ERS, but the role of ERS in NIHL still requires further research for clarification.

To sum up the above, hearing loss caused by genetics, drugs, noise and aging is related to ER homeostasis in inner ear cells, and furthermore, most studies have shown that SNHL is positively correlated with ERS. It is important to note that studies still lack evidence of a direct causal relationship between SNHL and ERS. Although the specific mechanism remains uncertain, ERS shows a strong correlation with SNHL. Thus, it appears that ERS has considerable potentiality for the study of mechanisms underlying SNHL and provides new potential targets for the treatment.

## 4 Potential targets and strategies against ERS for SNHL

**Figure 4:**
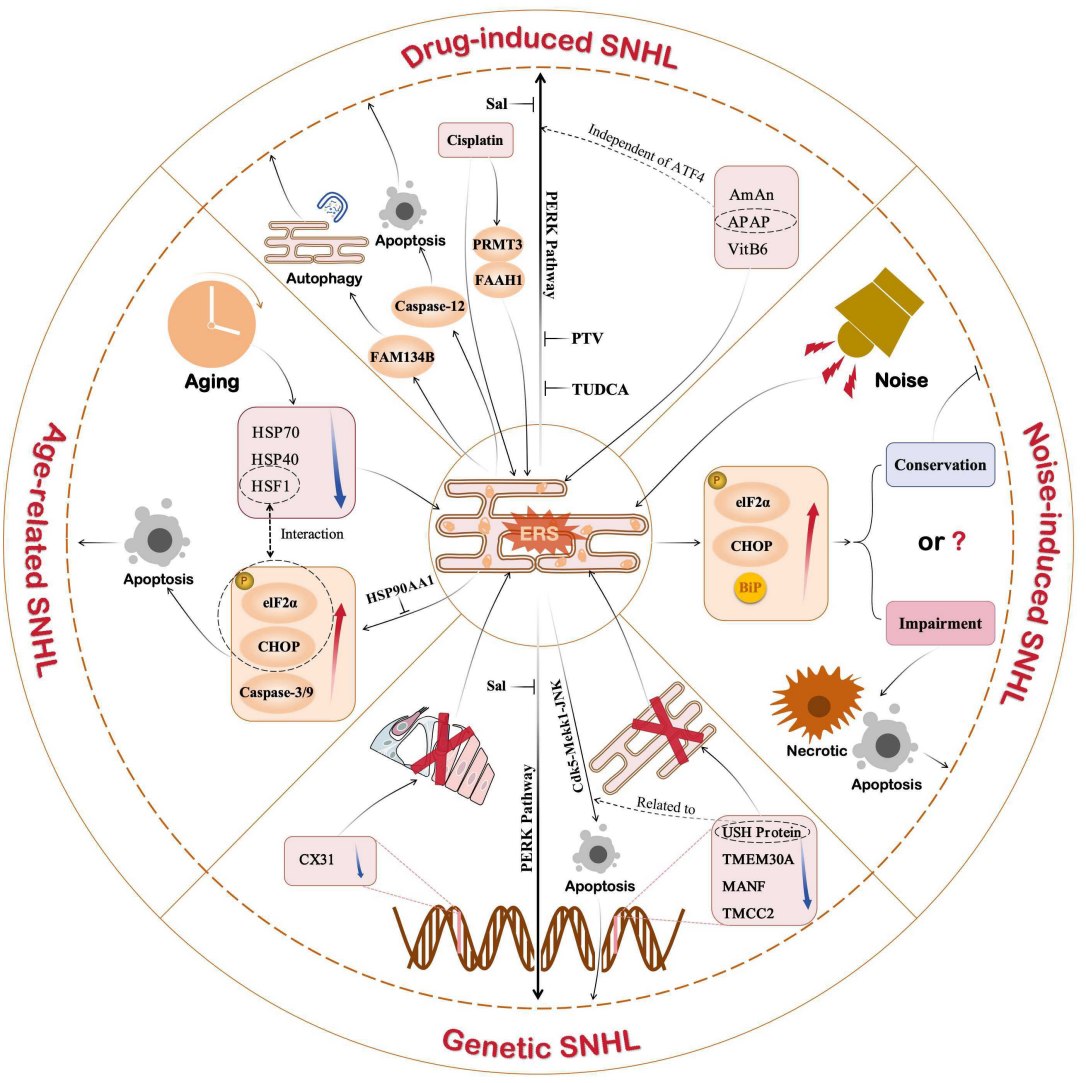
Role of ERS in different types of SNHL. Current studies show that ERS is associated with drug-induced, noise-induced, age-induced, or genetic SNHL. In addition to noise-induced SNHL, ERS shows a strong correlation with SNHL. Of the three UPR pathways, the PERK pathway plays an important role in SNHL. Therefore, drugs targeting the PERK pathway, such as Sal, PTV, and TUDCA, are promising new ideas for SNHL.

In recent years, scientists and clinicians have conducted numerous studies on ERS-mediated mechanisms of SNHL to identify therapeutic measures that target the mechanism underlying activation of the ERS response (Figure 4). The UPR is an important pathway in ERS-induced SNHL, in which the PERK/eIF2α/ATF4 pathway plays a central role. Several novel treatment strategies for SNHL have been proposed (Table 1). Salubrinal (Sal), a selective protein phosphatase I complex inhibitor, reduces HCs apoptosis in erl mice by inhibiting expression of BiP, CHOP, and caspase-3 in OHCs, SGNs, and stria vascularis (Hu et al., 2016). Thus, Sal is a promising option for patients with early DFNB12 and USH. Sal also inhibits cisplatin-induced cochlear cell apoptosis by dephosphorylating eIF2α, and by decreasing BiP and CHOP expression (Lu et al., 2022). Pitavastatin (PTV), a new-generation lipophilic statin, attenuates neomycin-induced ototoxicity by reducing the phosphorylation of PERK and eIF2α (Wu et al., 2022). Meanwhile, *in vivo* and *in vitro* experiments show that PTV reduces the expression of GRP78 and CHOP to suppress neomycin-induced ERS (Wu et al., 2022). Taurine ursodeoxycholic acid (TUDCA) is a co-cholestatic acid. Researchers found that the addition of TUDCA to gentamicin-induced cochlear explants alleviates ERS by inhibiting overexpression of BiP, CHOP, and caspase 3 (Jia et al., 2018). Studies also show that TUDCA reduces cisplatin-induced SNHL by increasing the efficacy of UFP/MFP processing in the ER (Wen et al., 2021). In 2023, Sun et al. conducted single-cell transcription experiments on cells from the cochlea and found that: (1) loss of protein homeostasis and apoptosis are the hallmark of ARHL; and (2) upregulation of the ER chaperone protein, HSP90AA1, alleviates damage to the cochlea caused by aging (Sun et al., 2023). Interestingly, it is the UPR that connects the loss of protein homeostasis with apoptosis. This suggests that ERS-induced apoptosis via UPR has enormous research potential in the context of ARHL. Inhibiting the apoptosis mediated by maladaptive UPR could be a novel treatment option for ARHL.

**Table 1:** Potential targets and treatment strategies for ERS-mediated SNHL.

**Target**	**Subject**	**Inner ear cells**	**Strategy**	**Inducement of HL**	**References**
CHOP	HEI-OC1 cells	—	Knockdown of *chop*	Cisplatin	Qu et al., 2023
	erl mice	OHCs, SGNs and stria vascularis	Sal	DFNB12 and USH	Hu et al., 2016
	C57BL/6 mice, HEI-OC1 cells	Cochlear HCs	PTV	Neomycin	Wu et al., 2022
	Cochlear explants	–	TUDCA	Gentamycin	Jia et al., 2018
BiP	erl mice	OHCs, SGNs and stria vascularis	Sal	DFNB12 and USH	Hu et al., 2016
	C57BL/6 mice, HEI-OC1 cells	Cochlear HCs	PTV	Neomycin	Wu et al., 2022
	Cochlear explants	—	TUDCA	Gentamycin	Jia et al., 2018
PERK	HEI-OC1 cells	—	PTV	Neomycin	Wu et al., 2022
eIF2α	Mouse cochlear explants and HEI-OC1 cells	—	Sal	Cisplatin	Lu et al., 2022
	HEI-OC1 cells	—	PTV	Neomycin	Wu et al., 2022
ATF4	OC-1 cells	—	Knockdown of *chop*	Cisplatin	Qu et al., 2023
Caspase-3	erl mice	OHCs, SGNs and stria vascularis	Sal	DFNB12 and USH	Hu et al., 2016
	Cochlear explants	—	TUDCA	Gentamycin	Jia et al., 2018
PRMT3	HEI-OC1 cells	—	Knockdown of PRMT3	Cisplatin	Lim et al., 2019
CB1R, FAAH1	HEI-OC1 cells	—	CB1R agonist, FAAH1 inhibitor	Cisplatin	Lim et al., 2019
HSP90AA1	Comprehensive single-cell transcriptomic atlas of mouse cochlear aging at high-temporal resolution	Stria vascularis	Active endogenous HSP90AA1	Age	Sun et al., 2023
FAM134B	HEI-OC1 cells	—	Knockdown of FAM134B	Cisplatin	Yang et al., 2023

## 5 Conclusions and future prospects

ER stress is a ubiquitous etiological mechanism involved in a variety of disease processes. Under the influence of genetic and environmental factors, ERS participates in the pathogenesis of SNHL by regulating cell function and fate through extensive communication with the biological processes behind gene expression, oxidative stress, apoptosis, inflammation and autophagy. An increasing amount of research has elucidated that ERS-induced cell death in the inner ear is essential in SNHL, suggesting a positive correlation between ERS and SNHL; however, the specific causal relationship between the two has yet to be clarified. There still remain several unclear questions, such as which cells in the inner ear are mainly affected by ERS and through which specific pathway does the UPR cause damage to the inner ear? Even though numerous medications appear to reduce ERS, they have only been tested in animals and cells, and there remains a notable lack of clinical trials. To sum up, ERS is a novel therapeutic focus in the context of SNHL and has tremendous research potential. The question of how to balance the protective and disease-causing arms of the UPR needs to be considered when identifying/designing new drugs for SNHL treatment. All abbreviations of specialized terminology included in this article are summarized in a table (Table 2).

**Table 2:** List of abbreviations.

**Abbreviation**	**Full name**
ABR	Auditory brainstem response
AARE	Amino acid response element
AmAn	Aminoglycoside antibiotic
APAP	Acetaminophen
ARHL	Age-related hearing loss
ASK1	Apoptosis signal-regulated kinase 1
ATF4	Activating transcription factor 4
ATF6	Activating transcription factor 6
ATG5	autophagy-regulated gene 5
Bcl-2	B cell lymphoma 2
Bcl-xL	B-cell lymphoma-extra large
CB1R	Cannabinoid 1 receptor
CHOP	C/EBP- homologous protein
DFNB12	Nonsyndromic autosomal recessive deafness
eIF2α	Eukaryotic translation initiation factor 2 subunit-α
ER	Endoplasmic reticulum
ERAD	ER-associated degradation
ERO1α	Endoplasmic reticulum oxidoreductase 1α
ERS	Endoplasmic reticulum stress
FoxO1	Forkhead box O1
GADD34	growth arrest and DNA damage-inducible 34
HC	Hair cell
HL	Hearing loss
IP3R	Inositol 1,4,5-triphosphate receptor type 3
IRE1α	Inositol-requiring enzyme 1α
JNK	C-Jun N-terminal kinase
MANF	Mesencephalic astrocyte-derived neurotrophic factor
MAP1LC3B	Microtubule-associated protein 1 light chain 3β
NAPQI	N-acetyl-P-benzoquinone imine
NF-κB	Nuclear factor- κB
NIHL	Noise-induced hearing loss
NRF2	Nuclear factor erythroid 2-related factor
OHCs	Outer hair cells
PERK	Pancreatic endoplasmic reticulum kinase
PRMT3	Protein arginine methyltransferase 3
PTV	Pitavastatin
RIDD	Regulated IRE1-dependent decay
RIPK1	Receptor-interacting serine/threonine-protein kinase 1
ROS	Reactive oxygen species
S1P	Site-1 protease
S2P	Site-2 protease
Sal	Salubrinal
SGNs	spiral ganglion neurons
SNHL	Sensorineural hearing loss
TMEM30A	Transmembrane protein 30A
TUDCA	Taurine ursodeoxycholic acid
UFP/MFP	Unfolded proteins/Misfolded proteins
UPR	Unfolded protein response
USH	Usher syndrome
USH1D	Usher syndrome type 1D
XBP1	X box binding protein 1

## References

[B1] Bao J. Ohlemiller K. K. (2010). Age-related loss of spiral ganglion neurons. Hear Res. 264, 93–97. 10.1016/j.heares.2009.10.009 19854255 PMC2868093

[B2] B'chir W. Maurin A.-C. Carraro V. Averous J. Jousse C. Muranishi Y.. (2013). The eIF2alpha/ATF4 pathway is essential for stress-induced autophagy gene expression. Nucleic Acids Res. 41, 7683–7699. 10.1093/nar/gkt563 23804767 PMC3763548

[B3] Blais J. D. Filipenko V. Bi M. Harding H. P. Ron D. Koumenis C.. (2004). Activating transcription factor 4 is translationally regulated by hypoxic stress. Mol. Cell Biol. 24, 7469–7482. 10.1128/MCB.24.17.7469-7482.2004 15314157 PMC506979

[B4] Blakley B. W. Schilling H. (2008). Deafness associated with acetaminophen and codeine abuse. J. Otolaryngol. Head Neck Surg. 37, 507–509. 10.2310/7070.2008.0096 19128584

[B5] Blanco-Sánchez B. Clément A. Jr. J. F. Washbourne P. Westerfield M. (2014). Complexes of Usher proteins preassemble at the endoplasmic reticulum and are required for trafficking and ER homeostasis. Dis. Model Mech. 7, 547–559. 10.1242/dmm.014068 24626987 PMC4007406

[B6] Bolz H. von Brederlow B. Ramírez A. Bryda E. C. Kutsche K. Nothwang H. G.. (2001). Mutation of CDH23, encoding a new member of the cadherin gene family, causes Usher syndrome type 1D. Nat. Genet. 27, 108–112. 10.1038/83667 11138009

[B7] Bonnet C. El-Amraoui A. (2012). Usher syndrome (sensorineural deafness and retinitis pigmentosa): pathogenesis, molecular diagnosis and therapeutic approaches. Curr. Opin. Neurol. 25, 42–49. 10.1097/WCO.0b013e32834ef8b2 22185901

[B8] Bork J. M. Peters L. M. Riazuddin S. Bernstein S. L. Ahmed Z. M. Ness S. L.. (2001). Usher syndrome 1D and nonsyndromic autosomal recessive deafness DFNB12 are caused by allelic mutations of the novel cadherin-like gene CDH23. Am. J. Hum. Genet. 68, 26–37. 10.1086/316954 11090341 PMC1234923

[B9] Brüning A. Rahmeh M. Friese K. (2013). Nelfinavir and bortezomib inhibit mTOR activity via ATF4-mediated sestrin-2 regulation. Mol. Oncol. 7, 1012–1018. 10.1016/j.molonc.2013.07.010 23916134 PMC5528439

[B10] Calfon M. Zeng H. Urano F. Till J. H. Hubbard S. R. Harding H. P.. (2002). IRE1 couples endoplasmic reticulum load to secretory capacity by processing the XBP-1 mRNA. Nature 415, 92–96. 10.1038/415092a 11780124

[B11] Carpena N. T. Lee M. Y. (2018). Genetic hearing loss and gene therapy. Genom. Inform. 16:e20. 10.5808/GI.2018.16.4.e20 30602081 PMC6440668

[B12] Chen B. L. (2015). CCAAT-enhancer-binding protein homologous protein deficiency attenuates oxidative stress and renal ischemia-reperfusion injury. Antioxid Redox Signal 23, 1233–1245. 10.1089/ars.2013.5768 25178318

[B13] Chen Y. Brandizzi F. (2013). IRE1: ER stress sensor and cell fate executor. Trends Cell Biol. 23, 547–555. 10.1016/j.tcb.2013.06.005 23880584 PMC3818365

[B14] Chepurwar S. von Loh S. M. Wigger D. C. Neef J. Frommolt P. Beutner D.. (2023). A mutation in ATP11A causes autosomal-dominant auditory neuropathy type 2. Hum. Mol. Genet. 32, 1083–1089. 10.1093/hmg/ddac267 36300302

[B15] Cohen-Salmon M. Ott T. Michel V. Hardelin J. P. Perfettini I. Eybalin M.. (2002). Targeted ablation of connexin26 in the inner ear epithelial gap junction network causes hearing impairment and cell death. Curr. Biol. 12, 1106–1111. 10.1016/S0960-9822(02)00904-1 12121617 PMC4030438

[B16] Coleman J. A. Kwok M. C. Molday R. S. (2009). Localization, purification, and functional reconstitution of the P4-ATPase Atp8a2, a phosphatidylserine flippase in photoreceptor disc membranes. J. Biol. Chem. 284, 32670–32679. 10.1074/jbc.M109.047415 19778899 PMC2781682

[B17] Coleman J. A. Zhu X. Djajadi H. R. Molday L. L. Smith R. S. Libby R. T.. (2014). A phospholipid flippase ATP8A2 is required for normal visual and auditory function and photoreceptor and spiral ganglion cell survival. J. Cell Sci. 127, 1138–1149. 10.1242/jcs.145052 24413176 PMC3937779

[B18] Cullinan S. B. Diehl J. A. (2004). PERK-dependent activation of Nrf2 contributes to redox homeostasis and cell survival following endoplasmic reticulum stress. J. Biol. Chem. 279, 20108–20117. 10.1074/jbc.M314219200 14978030

[B19] Cullinan S. B. Zhang D. Hannink M. Arvisais E. Kaufman R. J. Diehl J. A. (2003). Nrf2 is a direct PERK substrate and effector of PERK-dependent cell survival. Mol. Cell Biol. 23, 7198–7209. 10.1128/MCB.23.20.7198-7209.2003 14517290 PMC230321

[B20] Curhan S. G. Eavey R. Shargorodsky J. Curhan G. C. (2010). Analgesic use and the risk of hearing loss in men. Am. J. Med. 123, 231–237. 10.1016/j.amjmed.2009.08.006 20193831 PMC2831770

[B21] Curhan S. G. Shargorodsky J. Eavey R. Curhan G. C. (2012). Analgesic use and the risk of hearing loss in women. Am. J. Epidemiol. 176, 544–554. 10.1093/aje/kws146 22933387 PMC3530351

[B22] Cybulsky A. V. (2017). Endoplasmic reticulum stress, the unfolded protein response and autophagy in kidney diseases. Nat. Rev. Nephrol. 13, 681–696. 10.1038/nrneph.2017.129 28970584

[B23] Deng J. Lu P. D. Zhang Y. Scheuner D. Kaufman R. J. Sonenberg N.. (2004). Translational repression mediates activation of nuclear factor kappa B by phosphorylated translation initiation factor 2. Mol. Cell Biol. 24, 10161–10168. 10.1128/MCB.24.23.10161-10168.2004 15542827 PMC529034

[B24] Duran-Aniotz C. Cornejo V. H. Espinoza S. Ardiles Á. O. Medinas D. B. Salazar C.. (2017). IRE1 signaling exacerbates Alzheimer's disease pathogenesis. Acta Neuropathol. 134, 489–506. 10.1007/s00401-017-1694-x 28341998

[B25] Eggermont J. J. Profant O. Tintěra J. Svobodová V. Tóthová D. Škoch A.. (2017). Epidemiology and genetics of hearing loss and tinnitus. Hear. Loss 4, 209–234. 10.1016/B978-0-12-805398-0.00007-4

[B26] Fedoroff N. (2006). Redox regulatory mechanisms in cellular stress responses. Ann. Bot. 98, 289–300. 10.1093/aob/mcl128 16790465 PMC2803466

[B27] Feldman D. E. Chauhan V. Koong A. C. (2005). The unfolded protein response: a novel component of the hypoxic stress response in tumors. Mol. Cancer Res. 3, 597–605. 10.1158/1541-7786.MCR-05-0221 16317085

[B28] Ferguson M. A. Kitterick P. T. Chong L. Y. Edmondson-Jones M. Barker F. Hoare D. J. (2017). Hearing aids for mild to moderate hearing loss in adults. Cochr. Datab. Syst. Rev. 9:CD012023. 10.1002/14651858.CD012023.pub2 28944461 PMC6483809

[B29] Fonseca S. G. Gromada J. Urano F. (2011). Endoplasmic reticulum stress and pancreatic β-cell death. Trends Endocrinol. Metab. 22, 266–274. 10.1016/j.tem.2011.02.008 21458293 PMC3130122

[B30] Francis S. P. Katz J. Fanning K. D. Harris K. A. Nicholas B. D. Lacy M.. (2013). A novel role of cytosolic protein synthesis inhibition in aminoglycoside ototoxicity. J. Neurosci. 33, 3079–3093. 10.1523/JNEUROSCI.3430-12.2013 23407963 PMC3711767

[B31] Fuente A. Hickson L. (2011). Noise-induced hearing loss in Asia. Int. J. Audiol. 50(Suppl.1), S3–S10. 10.3109/14992027.2010.540584 21288065

[B32] Gao Z. Liu G. Hu Z. Li X. Yang X. Jiang B.. (2014). Grape seed proanthocyanidin extract protects from cisplatin-induced nephrotoxicity by inhibiting endoplasmic reticulum stress-induced apoptosis. Mol. Med. Rep. 9, 801–807. 10.3892/mmr.2014.1883 24399449 PMC3926513

[B33] Gentilin E. Simoni E. Candito M. Cazzador D. Astolfi L. (2019). Cisplatin-induced ototoxicity: updates on molecular targets. Trends Mol. Med. 25, 1123–1132. 10.1016/j.molmed.2019.08.002 31473143

[B34] Giraudet F. Charles P. Mom T. Boespflug-Tanguy O. Dürr A. Deltenre P.. (2018). Rapid exhaustion of auditory neural conduction in a prototypical mitochondrial disease, Friedreich ataxia. Clin. Neurophysiol. 129, 1121–1129. 10.1016/j.clinph.2018.03.005 29625343

[B35] Glembotski C. C. Thuerauf D. J. Huang C. Vekich J. A. Gottlieb R. A. Doroudgar S. (2012). Mesencephalic astrocyte-derived neurotrophic factor protects the heart from ischemic damage and is selectively secreted upon sarco/endoplasmic reticulum calcium depletion. J. Biol. Chem. 287, 25893–25904. 10.1074/jbc.M112.356345 22637475 PMC3406674

[B36] Goodall J. C. Wu C. Zhang Y. McNeill L. Ellis L. Saudek V.. (2010). Endoplasmic reticulum stress-induced transcription factor, CHOP, is crucial for dendritic cell IL-23 expression. Proc. Natl. Acad. Sci. U. S. A. 107, 17698–17703. 10.1073/pnas.1011736107 20876114 PMC2955096

[B37] Görlach A. Klappa P. Kietzmann T. (2006). The endoplasmic reticulum: folding, calcium homeostasis, signaling, and redox control. Antioxid Redox Signal. 8, 1391–1418. 10.1089/ars.2006.8.1391 16986999

[B38] Gu S. Chen C. Jiang X. Zhang Z. (2016). ROS-mediated endoplasmic reticulum stress and mitochondrial dysfunction underlie apoptosis induced by resveratrol and arsenic trioxide in A549 cells. Chem. Biol. Interact. 245, 100–109. 10.1016/j.cbi.2016.01.005 26772155

[B39] Guo L. Cao W. Niu Y. He S. Chai R. Yang J. (2021). Autophagy regulates the survival of hair cells and spiral ganglion neurons in cases of noise, ototoxic drug, and age-induced sensorineural hearing loss. Front. Cell Neurosci. 15:760422. 10.3389/fncel.2021.760422 34720884 PMC8548757

[B40] Han F. Yu H. Tian C. Chen H. E. Benedict-Alderfer C. Zheng Y.. (2012). A new mouse mutant of the Cdh23 gene with early-onset hearing loss facilitates evaluation of otoprotection drugs. Pharmacogenom. J. 12, 30–44. 10.1038/tpj.2010.60 20644563 PMC3000876

[B41] Han J. Back S. H. Hur J. Lin Y. H. Gildersleeve R. Shan J.. (2013). ER-stress-induced transcriptional regulation increases protein synthesis leading to cell death. Nat. Cell Biol. 15, 481–490. 10.1038/ncb2738 23624402 PMC3692270

[B42] Harding H. P. Zhang Y. Ron D. (1999). Protein translation and folding are coupled by an endoplasmic-reticulum-resident kinase. Nature 397, 271–274. 10.1038/16729 9930704

[B43] Haze K. Yoshida H. Yanagi H. Yura T. Mori K (1999). Mammalian transcription factor ATF6 is synthesized as a transmembrane protein and activated by proteolysis in response to endoplasmic reticulum stress. Mol. Biol. Cell. 10, 3787–3799. 10.1091/mbc.10.11.3787 10564271 PMC25679

[B44] He Z. Guo L. Shu Y. Fang Q. Zhou H. Liu Y.. (2017). Autophagy protects auditory hair cells against neomycin-induced damage. Autophagy 13, 1884–1904. 10.1080/15548627.2017.1359449 28968134 PMC5788479

[B45] He Z.-H. Li M. Fang Q.-J. Liao F.-L. Zou S.-Y. Wu X.. (2021). FOXG1 promotes aging inner ear hair cell survival through activation of the autophagy pathway. Autophagy 17, 4341–4362. 10.1080/15548627.2021.1916194 34006186 PMC8726647

[B46] Heath-Engel H. M. Chang N. C. Shore G. C. (2008). The endoplasmic reticulum in apoptosis and autophagy: role of the BCL-2 protein family. Oncogene 27, 6419–6433. 10.1038/onc.2008.309 18955970

[B47] Herranen A. Ikäheimo K. Lankinen T. Pakarinen E. Fritzsch B. Saarma M.. (2020). Deficiency of the ER-stress-regulator MANF triggers progressive outer hair cell death and hearing loss. Cell Death Dis. 11:100. 10.1038/s41419-020-2286-6 32029702 PMC7005028

[B48] Hetz C. Chevet E. Oakes S. A. (2015). Proteostasis control by the unfolded protein response. Nat. Cell Biol. 17, 829–838. 10.1038/ncb3184 26123108 PMC5546321

[B49] Hetz C. Zhang K. Kaufman R. J. (2020). Mechanisms, regulation and functions of the unfolded protein response. Nat. Rev. Mol. Cell Biol. 21, 421–438. 10.1038/s41580-020-0250-z 32457508 PMC8867924

[B50] Hopkins P. C. Sáinz-Fuertes R. Lovestone S. (2011). The impact of a novel apolipoprotein E and amyloid-β protein precursor-interacting protein on the production of amyloid-β. J. Alzheimer's Dis. 26, 239–253. 10.3233/JAD-2011-102115 21593558

[B51] Hoyer M. J. Chitwood P. J. Ebmeier C. C. Striepen J. F. Qi R. Z. Old W. M.. (2018). Novel class of ER membrane proteins regulates ER-associated endosome fission. Cell 175, 254-265.e14. 10.1016/j.cell.2018.08.030 30220460 PMC6195207

[B52] Hu J. Li B. Apisa L. Yu H. Entenman S. Xu M.. (2016). ER stress inhibitor attenuates hearing loss and hair cell death in Cdh23(erl/erl) mutant mice. Cell Death Dis. 7:e2485. 10.1038/cddis.2016.386 27882946 PMC5260868

[B53] Iurlaro R. Muñoz-Pinedo C. (2016). Cell death induced by endoplasmic reticulum stress. FEBS J. 283, 2640–2652. 10.1111/febs.13598 26587781

[B54] Jia Z. He Q. Shan C. Li F. (2018). Tauroursodeoxycholic acid attenuates gentamicin-induced cochlear hair cell death *in vitro*. Toxicol. Lett. 294, 20–26. 10.1016/j.toxlet.2018.05.007 29751043

[B55] Jin H.-O. Seo S.-K. Woo S.-H. Kim E.-S. Lee H.-C. Yoo D.-H.. (2009). Activating transcription factor 4 and CCAAT/enhancer-binding protein-beta negatively regulate the mammalian target of rapamycin via Redd1 expression in response to oxidative and endoplasmic reticulum stress. Free Radic. Biol. Med. 46, 1158–1167. 10.1016/j.freeradbiomed.2009.01.015 19439225

[B56] Jin J.-K. Blackwood E. A. Azizi K. Thuerauf D. J. Fahem A. G. Hofmann C.. (2017). ATF6 decreases myocardial ischemia/reperfusion damage and links ER stress and oxidative stress signaling pathways in the heart. Circ. Res. 120, 862–875. 10.1161/CIRCRESAHA.116.310266 27932512 PMC5336510

[B57] Kalinec G. M. Thein P. Parsa A. Yorgason J. Luxford W. Urrutia R.. (2014). Acetaminophen and NAPQI are toxic to auditory cells via oxidative and endoplasmic reticulum stress-dependent pathways. Hear Res. 313, 26–37. 10.1016/j.heares.2014.04.007 24793116 PMC4084927

[B58] Kang M. J. Chung J. Ryoo H. D. (2012). CDK5 and MEKK1 mediate pro-apoptotic signalling following endoplasmic reticulum stress in an autosomal dominant retinitis pigmentosa model. Nat Cell Biol. 14, 409–415. 10.1038/ncb2447 22388889 PMC3319494

[B59] Kaufman R. J. (2002). Orchestrating the unfolded protein response in health and disease. J. Clin. Invest. 110, 1389–1398. 10.1172/JCI0216886 12438434 PMC151822

[B60] Kaufman R. J. Scheuner D. Schröder M. Shen X. Lee K. Liu C. Y.. (2002). The unfolded protein response in nutrient sensing and differentiation. Nat. Rev. Mol. Cell Biol. 3, 411–421. 10.1038/nrm829 12042763

[B61] Kawasaki N. Asada R. Saito A. Kanemoto S. Imaizumi K. (2012). Obesity-induced endoplasmic reticulum stress causes chronic inflammation in adipose tissue. Sci. Rep. 2:799. 10.1038/srep00799 23150771 PMC3495279

[B62] Kishino A. Hayashi K. Hidai C. Masuda T. Nomura Y. Oshima T. (2017). XBP1-FoxO1 interaction regulates ER stress-induced autophagy in auditory cells. Sci. Rep. 7:4442. 10.1038/s41598-017-02960-1 28667325 PMC5493624

[B63] Kishino A. Hayashi K. Maeda M. Jike T. Hidai C. Nomura Y.. (2019). Caspase-8 regulates endoplasmic reticulum stress-induced necroptosis independent of the apoptosis pathway in auditory cells. Int. J. Mol. Sci. 20:235896. 10.3390/ijms20235896 31771290 PMC6928907

[B64] Kong D. Zhuo L. Gao C. Shi S. Wang N. Huang Z.. (2013). Erythropoietin protects against cisplatin-induced nephrotoxicity by attenuating endoplasmic reticulum stress-induced apoptosis. J. Nephrol. 26, 219–227. 10.5301/jn.5000177 22711436

[B65] Kujawa S. G. Liberman M. C. (2015). Synaptopathy in the noise-exposed and aging cochlea: primary neural degeneration in acquired sensorineural hearing loss. Hear Res. 330, 191–199. 10.1016/j.heares.2015.02.009 25769437 PMC4567542

[B66] Lang H. Schulte B. A. Schmiedt R. A. (2005). Ouabain induces apoptotic cell death in type I spiral ganglion neurons, but not type II neurons. J. Assoc. Res. Otolaryngol. 6, 63–74. 10.1007/s10162-004-5021-6 15735933 PMC2504640

[B67] Lee Y. Y. Gil E. S. Jeong I. H. Kim H. Jang J. H. Choung Y.-H. (2021). Heat shock factor 1 prevents age-related hearing loss by decreasing endoplasmic reticulum stress. Cells 10:92454. 10.3390/cells10092454 34572102 PMC8468389

[B68] Li C.-M. Zhang X. Hoffman H. J. Cotch M. F. Themann C. L. Wilson M. R. (2014). Hearing impairment associated with depression in US adults, National Health and Nutrition Examination Survey 2005-2010. J. Am. Med. Assoc. Otolaryngol. Head Neck Surg. 140, 293–302. 10.1001/jamaoto.2014.42 24604103 PMC4102382

[B69] Li J. Akil O. Rouse S. L. McLaughlin C. W. Matthews I. R. Lustig L. R.. (2018). Deletion of Tmtc4 activates the unfolded protein response and causes postnatal hearing loss. J. Clin. Invest. 128, 5150–5162. 10.1172/JCI97498 30188326 PMC6205388

[B70] Li W. Cao T. Luo C. Cai J. Zhou X. Xiao X.. (2020). Crosstalk between ER stress, NLRP3 inflammasome, and inflammation. Appl. Microbiol. Biotechnol. 104, 6129–6140. 10.1007/s00253-020-10614-y 32447438

[B71] Liberman M. C. (2017). Noise-induced and age-related hearing loss: new perspectives and potential therapies. F1000Res 6:927. 10.12688/f1000research.11310.1 28690836 PMC5482333

[B72] Lim J.-O. Ko J.-W. Shin N.-R. Jung T.-Y. Moon C. Kim H.-C.. (2019). Cisplatin-induced ototoxicity involves interaction of PRMT3 and cannabinoid system. Arch. Toxicol. 93, 2335–2346. 10.1007/s00204-019-02507-5 31256211

[B73] Liu W. Xu X. Fan Z. Sun G. Han Y. Zhang D.. (2019). Wnt signaling activates TP53-induced glycolysis and apoptosis regulator and protects against cisplatin-induced spiral ganglion neuron damage in the mouse cochlea. Antioxid Redox Signal 30, 1389–1410. 10.1089/ars.2017.7288 29587485

[B74] Livingston G. Huntley J. Sommerlad A. Ames D. Ballard C. Banerjee S.. (2020). Dementia prevention, intervention, and care: 2020 report of the Lancet Commission. Lancet 396, 413–446. 10.1016/S0140-6736(20)30367-6 32738937 PMC7392084

[B75] López-Bigas N. Olivé M. Rabionet R. Ben-David O. Martínez-Matos J. A. Bravo O.. (2001). Connexin 31 (GJB3) is expressed in the peripheral and auditory nerves and causes neuropathy and hearing impairment. Hum. Mol. Genet. 10, 947–952. 10.1093/hmg/10.9.947 11309368

[B76] Lu W. Ni K. Li Z. Xiao L. Li Y. Jiang Y.. (2022). Salubrinal protects against cisplatin-induced cochlear hair cell endoplasmic reticulum stress by regulating eukaryotic translation initiation factor 2α signalling. Front. Mol. Neurosci. 15:916458. 10.3389/fnmol.2022.916458 35706425 PMC9189388

[B77] Lv J. Fu X. Li Y. Hong G. Li P. Lin J.. (2021). Deletion of Kcnj16 in mice does not alter auditory function. Front. Cell Dev. Biol. 9:630361. 10.3389/fcell.2021.630361 33693002 PMC7937937

[B78] Ma K. Zhang Y. Zhao J. Zhou L. Li M. (2024). Endoplasmic reticulum stress: bridging inflammation and obesity-associated adipose tissue. Front. Immunol. 15:1381227. 10.3389/fimmu.2024.1381227 38638434 PMC11024263

[B79] Mandic A. Hansson J. Linder S. Shoshan M. C. (2003). Cisplatin induces endoplasmic reticulum stress and nucleus-independent apoptotic signaling. J. Biol. Chem. 278, 9100–9106. 10.1074/jbc.M210284200 12509415

[B80] Marciniak S. J. Yun C. Y. Oyadomari S. Novoa I. Zhang Y. Jungreis R.. (2004). CHOP induces death by promoting protein synthesis and oxidation in the stressed endoplasmic reticulum. Genes Dev. 18, 3066–3077. 10.1101/gad.1250704 15601821 PMC535917

[B81] Margariti A. Li H. Chen T. Martin D. Vizcay-Barrena G. Alam S.. (2013). XBP1 mRNA splicing triggers an autophagic response in endothelial cells through BECLIN-1 transcriptional activation. J. Biol. Chem. 288, 859–872. 10.1074/jbc.M112.412783 23184933 PMC3543035

[B82] Mener D. J. Betz J. Genther D. J. Chen D. Lin F. R. (2013). Hearing loss and depression in older adults. J. Am. Geriatr. Soc. 61, 1627–1629. 10.1111/jgs.12429 24028365 PMC3773611

[B83] Miyagawa M. Nishio S. Y. Usami S. (2012). Prevalence and clinical features of hearing loss patients with CDH23 mutations: a large cohort study. PLoS ONE 7:e40366. 10.1371/journal.pone.0040366 22899989 PMC3416829

[B84] Mizobuchi N. Hoseki J. Kubota H. Toyokuni S. Nozaki J.-i. Naitoh M.. (2007). ARMET is a soluble ER protein induced by the unfolded protein response via ERSE-II element. Cell Struct. Funct. 32, 41–50. 10.1247/csf.07001 17507765

[B85] Mo J. Chen J. Zhang B. (2020). Critical roles of FAM134B in ER-phagy and diseases. Cell Death Dis. 11:983. 10.1038/s41419-020-03195-1 33199694 PMC7670425

[B86] Nakagawa T. Zhu H. Morishima N. Li E. Xu J. Yankner B. A.. (2000). Caspase-12 mediates endoplasmic-reticulum-specific apoptosis and cytotoxicity by amyloid-beta. Nature 403, 98–103. 10.1038/47513 10638761

[B87] Ohoka N. Yoshii S. Hattori T. Onozaki K. Hayashi H. (2005). TRB3, a novel ER stress-inducible gene, is induced via ATF4-CHOP pathway and is involved in cell death. EMBO J. 24, 1243–1255. 10.1038/sj.emboj.7600596 15775988 PMC556400

[B88] Oishi N. Duscha S. Boukari H. Meyer M. Xie J. Wei G.. (2015). XBP1 mitigates aminoglycoside-induced endoplasmic reticulum stress and neuronal cell death. Cell Death Dis. 6:e1763. 10.1038/cddis.2015.108 25973683 PMC4669688

[B89] Omichi R. Shibata S. B. Morton C. C. Smith R. J. H. (2019). Gene therapy for hearing loss. Hum. Mol. Genet. 28, R65–R79. 10.1093/hmg/ddz129 31227837 PMC6796998

[B90] Park C. Lim H. Moon S. K. Park R. (2019). Pyridoxine preferentially induces auditory neuropathy through mitochondrial dysfunction and endoplasmic reticulum stress-mediated apoptosis. Ann. Otol. Rhinol. Laryngol. 128(6_Suppl.), 117S−124S. 10.1177/0003489419836116 31092035

[B91] Pater J. A. Penney C. O'Rielly D. D. Griffin A. Kamal L. Brownstein Z.. (2022). Autosomal dominant non-syndromic hearing loss maps to DFNA33 (13q34) and co-segregates with splice and frameshift variants in ATP11A, a phospholipid flippase gene. Hum. Genet. 141, 431–444. 10.1007/s00439-022-02444-x 35278131 PMC9035003

[B92] Petit C. Bonnet C. Safieddine S. (2023). Deafness: from genetic architecture to gene therapy. Nat. Rev. Genet. 24, 665–686. 10.1038/s41576-023-00597-7 37173518

[B93] Prasad V. Greber U. F. (2021). The endoplasmic reticulum unfolded protein response - homeostasis, cell death and evolution in virus infections. FEMS Microbiol. Rev. 45:fuab016. 10.1093/femsre/fuab016 33765123 PMC8498563

[B94] Qu Y. Zong S. Wang Z. Du P. Wen Y. Li H.. (2023). The PERK/ATF4/CHOP signaling branch of the unfolded protein response mediates cisplatin-induced ototoxicity in hair cells. Drug Chem. Toxicol. 46, 369–379. 10.1080/01480545.2022.2039181 35172660

[B95] Ramachandran A. Jaeschke H. (2018). Acetaminophen toxicity: novel insights into mechanisms and future perspectives. Gene Expr. 18, 19–30. 10.3727/105221617X15084371374138 29054140 PMC5885144

[B96] Ramming T. Okumura M. Kanemura S. Baday S. Birk J. Moes S.. (2015). PDI-catalyzed thiol-disulfide switch regulates the production of hydrogen peroxide by human Ero1. Free Radic. Biol. Med. 83, 361–372. 10.1016/j.freeradbiomed.2015.02.011 25697776

[B97] Rashid H. O. Yadav R. K. Kim H. R. Chae H. J. (2015). ER stress: autophagy induction, inhibition and selection. Autophagy 11, 1956–1977. 10.1080/15548627.2015.1091141 26389781 PMC4824587

[B98] Ren J. Bi Y. Sowers J. R. Hetz C. Zhang Y. (2021). Endoplasmic reticulum stress and unfolded protein response in cardiovascular diseases. Nat. Rev. Cardiol. 18, 499–521. 10.1038/s41569-021-00511-w 33619348

[B99] Ren R. Xing H. Wang X. Du H. Wang Y. Xu Z. (2023). Loss of TMCC2 activates endoplasm reticulum stress and causes auditory hair cell death. Hum. Mol. Genet. 32, 1622–1633. 10.1093/hmg/ddad003 36617157

[B100] Ron D. Walter P. (2007). Signal integration in the endoplasmic reticulum unfolded protein response. Nat. Rev. Mol. Cell Biol. 8, 519–529. 10.1038/nrm2199 17565364

[B101] Rouschop K. M. A. van den Beucken T. Dubois L. Niessen H. Bussink J. Savelkouls K.. (2010). The unfolded protein response protects human tumor cells during hypoxia through regulation of the autophagy genes MAP1LC3B and ATG5. J. Clin. Invest. 120, 127–141. 10.1172/JCI40027 20038797 PMC2798689

[B102] Saveljeva S. Mc Laughlin S. L. Vandenabeele P. Samali A. Bertrand M. J. M. (2015). Endoplasmic reticulum stress induces ligand-independent TNFR1-mediated necroptosis in L929 cells. Cell Death Dis. 6:e1587. 10.1038/cddis.2014.548 25569104 PMC4669746

[B103] Sawada N. Yao J. Hiramatsu N. Hayakawa K. Araki I. Takeda M.. (2008). Involvement of hypoxia-triggered endoplasmic reticulum stress in outlet obstruction-induced apoptosis in the urinary bladder. Lab. Invest. 88, 553–563. 10.1038/labinvest.2008.21 18347581

[B104] Schaumburg H. Kaplan J. Windebank A. Vick N. Rasmus S. Pleasure D.. (1983). Sensory neuropathy from pyridoxine abuse. A new megavitamin syndrome. N. Engl. J. Med. 309, 445–448. 10.1056/NEJM198308253090801 6308447

[B105] Schwarz D. S. Blower M. D. (2016). The endoplasmic reticulum: structure, function and response to cellular signaling. Cell Mol. Life Sci. 73, 79–94. 10.1007/s00018-015-2052-6 26433683 PMC4700099

[B106] Shen X. Ellis R. E. Lee K. Liu C. Y. Yang K. Solomon A.. (2001). Complementary signaling pathways regulate the unfolded protein response and are required for *C. elegans* development. Cell 107, 893–903. 10.1016/S0092-8674(01)00612-2 11779465

[B107] Shulman E. Belakhov V. Wei G. Kendall A. Meyron-Holtz E. G. Ben-Shachar D.. (2014). Designer aminoglycosides that selectively inhibit cytoplasmic rather than mitochondrial ribosomes show decreased ototoxicity: a strategy for the treatment of genetic diseases. J. Biol. Chem. 289, 2318–2330. 10.1074/jbc.M113.533588 24302717 PMC3900975

[B108] Simon A. M. Goodenough D. A. (1998). Diverse functions of vertebrate gap junctions. Trends Cell Biol. 8, 477–483. 10.1016/S0962-8924(98)01372-5 9861669

[B109] Smith J. A. Turner M. J. DeLay M. L. Klenk E. I. Sowders D. P. Colbert R. A. (2008). Endoplasmic reticulum stress and the unfolded protein response are linked to synergistic IFN-beta induction via X-box binding protein 1. Eur. J. Immunol. 38, 1194–1203. 10.1002/eji.200737882 18412159 PMC2838478

[B110] Sohn W. J. Kim J. Y. Kim D. Park J. A. Lee Y. Kwon H. J. (2016). Expression and characterization of transmembrane and coiled-coil domain family 3. BMB Rep. 49, 629–634. 10.5483/BMBRep.2016.49.11.151 27697108 PMC5346324

[B111] Stapelbroek J. M. Peters T. A. van Beurden D. H. A. Curfs J. H. A. J. Joosten A. Beynon A. J.. (2009). ATP8B1 is essential for maintaining normal hearing. Proc. Natl. Acad. Sci. U. S. A. 106, 9709–9714. 10.1073/pnas.0807919106 19478059 PMC2700994

[B112] Sun G. Zheng Y. Fu X. Zhang W. Ren J. Ma S.. (2023). Single-cell transcriptomic atlas of mouse cochlear aging. Protein Cell 14, 180–201. 10.1093/procel/pwac058 36933008 PMC10098046

[B113] Tabas I. Ron D. (2011). Integrating the mechanisms of apoptosis induced by endoplasmic reticulum stress. Nat. Cell Biol. 13, 184–190. 10.1038/ncb0311-184 21364565 PMC3107571

[B114] Tang X. Halleck M. S. Schlegel R. A. Williamson P. (1996). A subfamily of P-type ATPases with aminophospholipid transporting activity. Science 272, 1495–1497. 10.1126/science.272.5267.1495 8633245

[B115] Tone T. Nakayama K. Takatsu H. Shin H.-W. (2020). ATPase reaction cycle of P4-ATPases affects their transport from the endoplasmic reticulum. FEBS Lett. 594, 412–423. 10.1002/1873-3468.13629 31571211

[B116] Tu Y. Fan G. Sun H. Cai X. Kong W. (2019). Endoplasmic reticulum stress is involved in spiral ganglion neuron apoptosis following chronic kanamycin-induced deafness. Biosci. Rep. 39:1749. 10.1042/BSR20181749 30626727 PMC6592474

[B117] Urano F. Wang X. Bertolotti A. Zhang Y. Chung P. Harding H. P.. (2000). Coupling of stress in the ER to activation of JNK protein kinases by transmembrane protein kinase IRE1. Science 287, 664–666. 10.1126/science.287.5453.664 10650002

[B118] Valdés P. Mercado G. Vidal R. L. Molina C. Parsons G. Court F. A.. (2014). Control of dopaminergic neuron survival by the unfolded protein response transcription factor XBP1. Proc. Natl. Acad. Sci. U. S. A. 111, 6804–6809. 10.1073/pnas.1321845111 24753614 PMC4020088

[B119] Wang W. Sun Y. Chen S. Zhou X. Wu X. Kong W.. (2015). Impaired unfolded protein response in the degeneration of cochlea cells in a mouse model of age-related hearing loss. Exp. Gerontol. 70, 61–70. 10.1016/j.exger.2015.07.003 26173054

[B120] Wei Y. Sinha S. Levine B. (2008). Dual role of JNK1-mediated phosphorylation of Bcl-2 in autophagy and apoptosis regulation. Autophagy 4, 949–951. 10.4161/auto.6788 18769111 PMC2677707

[B121] Wen Y. Zong S. Liu T. Du P. Li H. Xiao H. (2021). Tauroursodeoxycholic acid attenuates cisplatin-induced ototoxicity by inhibiting the accumulation and aggregation of unfolded or misfolded proteins in the endoplasmic reticulum. Toxicology 453:152736. 10.1016/j.tox.2021.152736 33631298

[B122] Wisesa S. Yamamoto Y. Sakisaka T. (2019). TMCC3 localizes at the three-way junctions for the proper tubular network of the endoplasmic reticulum. Biochem. J. 476, 3241–3260. 10.1042/BCJ20190359 31696206

[B123] Wong A. C. Ryan A. F. (2015). Mechanisms of sensorineural cell damage, death and survival in the cochlea. Front. Aging Neurosci. 7:58. 10.3389/fnagi.2015.00058 25954196 PMC4404918

[B124] Wu H. Guo H. Liu H. Cui H. Fang J. Zuo Z.. (2020). Copper sulfate-induced endoplasmic reticulum stress promotes hepatic apoptosis by activating CHOP, JNK and caspase-12 signaling pathways. Ecotoxicol. Environ. Saf. 191:110236. 10.1016/j.ecoenv.2020.110236 32001424

[B125] Wu J. Ye J. Kong W. Zhang S. Zheng Y. (2020). Programmed cell death pathways in hearing loss: a review of apoptosis, autophagy and programmed necrosis. Cell Prolif. 53:e12915. 10.1111/cpr.12915 33047870 PMC7653260

[B126] Wu Y. Meng W. Guan M. Zhao X. Zhang C. Fang Q.. (2022). Pitavastatin protects against neomycin-induced ototoxicity through inhibition of endoplasmic reticulum stress. Front. Mol. Neurosci. 15:963083. 10.3389/fnmol.2022.963083 35992197 PMC9381809

[B127] Xia K. Ma H. Xiong H. Pan Q. Huang L. Wang D.. (2010). Trafficking abnormality and ER stress underlie functional deficiency of hearing impairment-associated connexin-31 mutants. Protein Cell. 1, 935–943. 10.1007/s13238-010-0118-7 21204020 PMC4875122

[B128] Xiao Z. Mingsheng Z. Li Y. Aimei W. (2016). The role of endoplasmic reticulum stres in cisplatin-induced apoptosis of mouse cochlear hair cel *in vitro*. J. Audiol. Speech Pathol. 24, 54–57. 10.3969/j.issn.1006-7299.2016.01.013

[B129] Xiaomeng W. Menghan Y. Zi W. Shen R. Rui Z. Wei L. (2020). Protective effect of the pretreatment with panaxadiol saponins on acetaminophen-induced experimental liver damaged mice. J. Jilin Agricult. Univ. 42, 638–645. 10.13327/j.jjlau.2020.6035

[B130] Xie J. Talaska A. E. Schacht J. (2011). New developments in aminoglycoside therapy and ototoxicity. Hear. Res. 281, 28–37. 10.1016/j.heares.2011.05.008 21640178 PMC3169717

[B131] Xing Y. Peng K. Yi Q. Yu D. Shi H. Yang G.. (2023). TMEM30A is essential for hair cell polarity maintenance in postnatal mouse cochlea. Cell Mol. Biol. Lett. 28, 23. 10.1186/s11658-023-00437-w 36959542 PMC10035192

[B132] Xue Q. Li C. Chen J. Guo H. Li D. Wu X. (2016). The protective effect of the endoplasmic reticulum stress-related factors BiP/GRP78 and CHOP/Gadd153 on noise-induced hearing loss in guinea pigs. Noise Health 18, 247–255. 10.4103/1463-1741.192481 27762253 PMC5187652

[B133] Yan Y. Rato C. Rohland L. Preissler S. Ron D. (2019). MANF antagonizes nucleotide exchange by the endoplasmic reticulum chaperone BiP. Nat. Commun. 10:541. 10.1038/s41467-019-08450-4 30710085 PMC6358605

[B134] Yang H. Yin H. Wang Y. Liu J. Guo L. Zhao H.. (2023). FAM134B-induced endoplasmic reticulum (ER)-phagy exacerbates cisplatin-insulted hair cell apoptosis: possible relation to excessive ER stress. Arch. Biochem. Biophys. 748:109766. 10.1016/j.abb.2023.109766 37813237

[B135] Yang X. Srivastava R. Howell S. H. Bassham D. C. (2016). Activation of autophagy by unfolded proteins during endoplasmic reticulum stress. Plant J. 85, 83–95. 10.1111/tpj.13091 26616142

[B136] Ye J. Rawson R. B. Komuro R. Chen X. Davé U. P. Prywes R.. (2000). stress induces cleavage of membrane-bound ATF6 by the same proteases that process SREBPs. Mol. Cell 6, 1355–1364. 10.1016/S1097-2765(00)00133-7 11163209

[B137] Yoshida H. Matsui T. Yamamoto A. Okada T. Mori K. (2001). XBP1 mRNA is induced by ATF6 and spliced by IRE1 in response to ER stress to produce a highly active transcription factor. Cell 107, 881–891. 10.1016/S0092-8674(01)00611-0 11779464

[B138] Yoshida H. Okada T. Haze K. Yanagi H. Yura T. Negishi M.. (2000). ATF6 activated by proteolysis binds in the presence of NF-Y (CBF) directly to the cis-acting element responsible for the mammalian unfolded protein response. Mol. Cell Biol. 20, 6755–6767. 10.1128/MCB.20.18.6755-6767.2000 10958673 PMC86199

[B139] Yücel S. S. Stelzer W. Lorenzoni A. Wozny M. Langosch D. Lemberg M. K. (2019). The metastable XBP1u transmembrane domain defines determinants for intramembrane proteolysis by signal peptide peptidase. Cell Rep. 26, 3087–3099.e11. 10.1016/j.celrep.2019.02.057 30865896

[B140] Zhang C. Kho Y. S. Wang Z. Chiang Y. T. Ng G. K. Shaw P. C.. (2014). Transmembrane and coiled-coil domain family 1 is a novel protein of the endoplasmic reticulum. PLoS ONE 9:e85206. 10.1371/journal.pone.0085206 24454821 PMC3891740

[B141] Zhang J. Guo J. Yang N. Huang Y. Hu T. Rao C. (2022). Endoplasmic reticulum stress-mediated cell death in liver injury. Cell Death Dis. 13:1051. 10.1038/s41419-022-05444-x 36535923 PMC9763476

[B142] Zhang L. Wang A. (2012). Virus-induced ER stress and the unfolded protein response. Front. Plant Sci. 3:293. 10.3389/fpls.2012.00293 23293645 PMC3531707

[B143] Zhang W. Shi Y. Oyang L. Cui S. Li S. Li J.. (2024). Endoplasmic reticulum stress-a key guardian in cancer. Cell Death Discov. 10:343. 10.1038/s41420-024-02110-3 39080273 PMC11289465

[B144] Zhou X. Graham T. R. (2009). Reconstitution of phospholipid translocase activity with purified Drs2p, a type-IV P-type ATPase from budding yeast. Proc. Natl. Acad. Sci. U. S. A. 106, 16586–16591. 10.1073/pnas.0904293106 19805341 PMC2757829

